# Supplementary material for: Changes in Age-Specific Pregnancy Prevalence Proportions over the Covid-19 Pandemic in Manicaland, Zimbabwe
Source: medRxiv. 2025 Sep 29:2025.09.23.25336434. Preprint. [Version 2] doi: 10.1101/2025.09.23.25336434 (PMC12622138; doi:10.1101/2025.09.23.25336434)
Supplement: Supplement 1 [file media-1.pdf]

Changes in Age-Specific Pregnancy Prevalence Proportions over the Covid-19 Pandemic in Manicaland, Zimbabwe

SUPPLEMENTARY MATERIAL

Figure S1 Household census and individual survey participation rates in the Manicaland General Population Survey, 2018 to 2023. Graph A: pre-Covid-19, graph B: mid-Covid-19, and graph C: late-Covid-19.

A

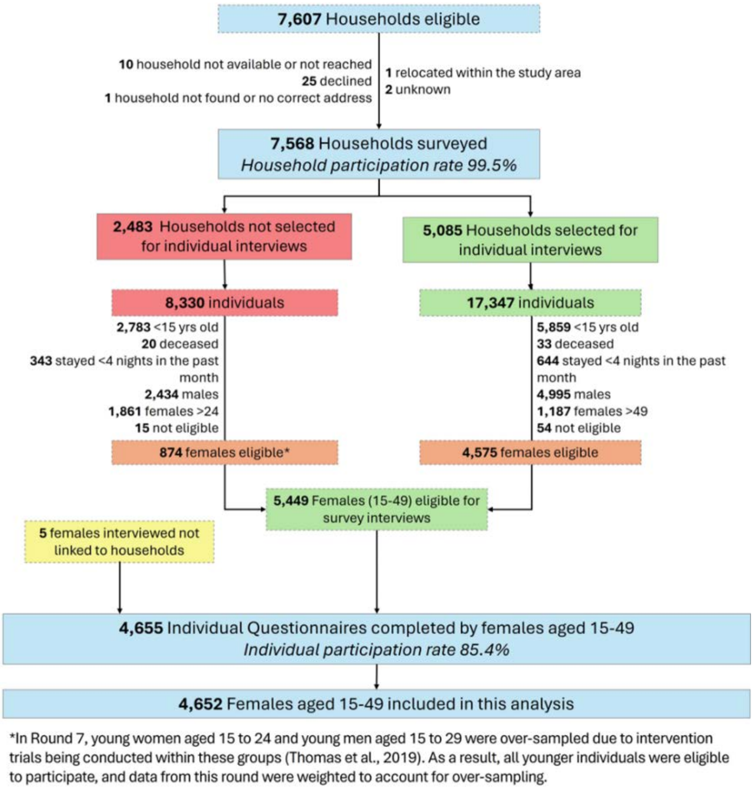

B

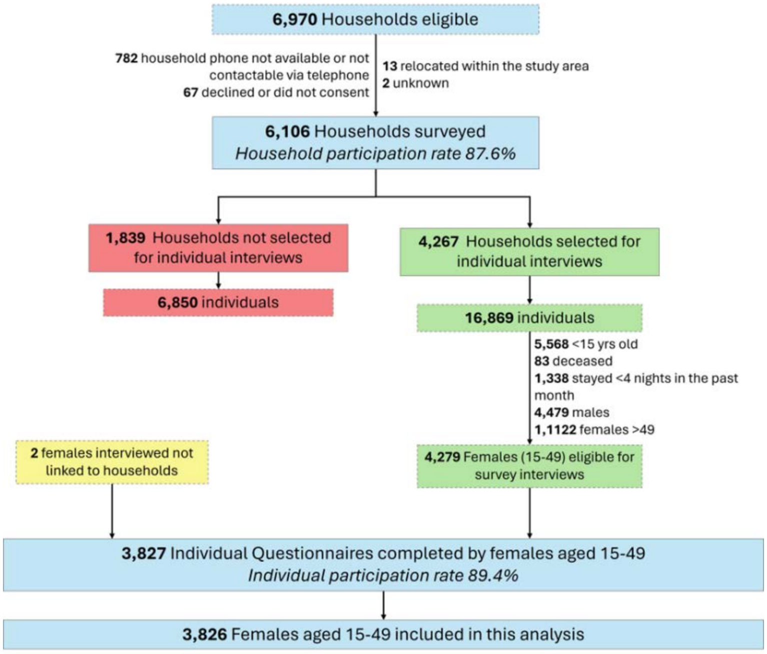
6,970 Households eligible
782 household phone not available or not contactable via telephone
67 declined or did not consent
13 relocated within the study area
2 unknown
6,106 Households surveyed
Household participation rate 87.6%
1,839 Households not selected for individual interviews
6,850 individuals
4,267 Households selected for individual interviews
16,869 individuals
5,568 <15 yrs old
83 deceased
1,338 stayed <4 nights in the past month
4,479 males
1,1122 females >49
2 females interviewed not linked to households
4,279 Females (15-49) eligible for survey interviews
3,827 Individual Questionnaires completed by females aged 15-49
Individual participation rate 89.4%
3,826 Females aged 15-49 included in this analysis

C

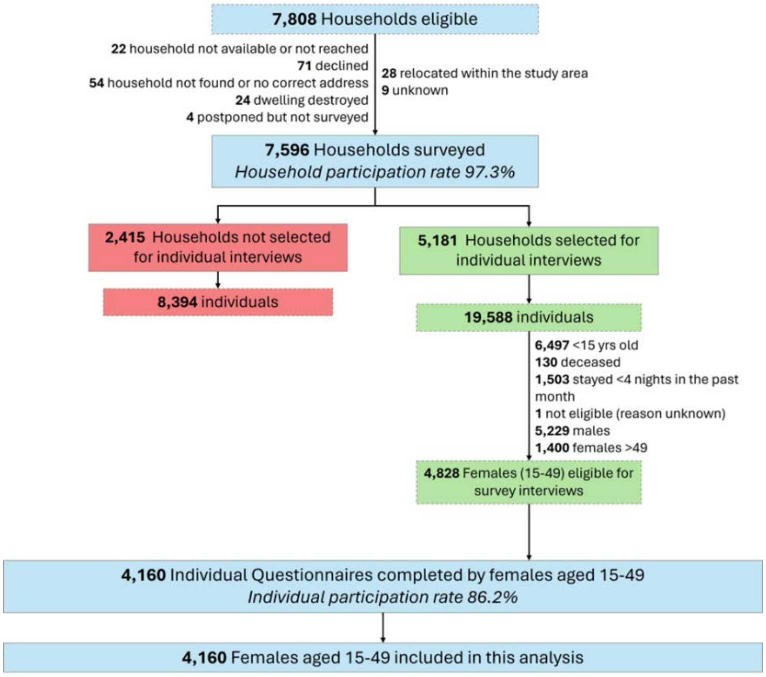
7,808 Households eligible
22 household not available or not reached
71 declined
54 household not found or no correct address
24 dwelling destroyed
4 postponed but not surveyed
28 relocated within the study area
9 unknown
7,596 Households surveyed
Household participation rate 97.3%
2,415 Households not selected for individual interviews
8,394 individuals
5,181 Households selected for individual interviews
19,588 individuals
6,497 <15 yrs old
130 deceased
1,503 stayed <4 nights in the past month
1 not eligible (reason unknown)
5,229 males
1,400 females >49
4,828 Females (15-49) eligible for survey interviews
4,160 Individual Questionnaires completed by females aged 15-49
Individual participation rate 86.2%
4,160 Females aged 15-49 included in this analysis

Table S1 Adjusted odds ratios of a recent birth in late- and mid- Covid-19 compared to before Covid-19, women aged 15-49 years, Manicaland, Zimbabwe

	Late-Covid-19 *vs.* Pre-Covid-19				Mid-Covid-19 *vs .* Pre-Covid-19				Pre-Covid-19	
Characteristic	AOR	*95% per cent CI*	%	*N*	AOR	*95% per cent CI*	%	*N*	%	*N*
All women	1.03	0.90-1.18	11.1	4,160	0.90	0.78-1.04	9.9	3,826	11.3	4652
Age-group										
15-19	0.77	0.52-1.13	5.3	795	0.74	0.49-1.11	4.9	753	6.7	1,167
20-24	1.01	0.79-1.30	18.2	714	0.95	0.73-1.23	17.4	588	18.1	1,049
25-34	1.16	0.93-1.45	17.9	1,105	0.92	0.73-1.16	15.1	1,054	16.2	1,148
35-49	1.03	0.75-1.41	5.8	1,546	0.98	0.71-1.35	5.7	1,431	5.9	1,288
Location										
Urban	0.84	0.63-1.12	10.6	1,045	0.63	0.45-0.88	8.0	717	12.7	893
Periurban	1.22	0.93-1.60	10.1	1,183	1.26	0.96-1.64	10.2	1,230	9.2	1,425
Agricultural estate	1.23	0.93-1.63	13.2	920	0.98	0.73-1.32	10.8	817	11.0	986
Rural	0.86	0.66-1.11	10.8	1,012	0.79	0.61-1.02	10.3	1,062	12.8	1,348
Marital status										
Single	0.57	0.29-1.13	1.2	966	0.92	0.49-1.74	1.8	875	2.1	1,265
Married	1.13	0.97-1.31	15.3	2,562	0.96	0.81-1.12	13.5	2,388	15.4	2,803
Divorced or separated	1.38	0.87-2.17	11.2	484	1.00	0.61-1.65	8.4	415	8.8	431
Widowed	0.19	0.03-1.29	0.7	148	1.05	0.27-4.13	3.4	148	3.5	153
Sexual activity										
Sexual debut	1.10	0.96-1.26	13.6	3,381	0.96	0.83-1.11	12.1	3,102	13.5	3679
Socio-economic status (quintile)										
Poorest	1.15	0.59-1.65	11.4	202	0.99	0.66-1.98	10.3	262	12.1	411
Second poorest	1.03	0.84-1.27	12.2	1,678	0.81	0.66-1.01	10.0	1,624	12.5	1,864
Third poorest	1.05	0.80-1.38	11.8	1,170	1.11	0.85-1.45	11.9	1,080	11.0	1,118
Fourth poorest	0.91	0.68-1.22	8.4	1,051	0.79	0.57-1.10	7.3	826	9.5	1,185
Least poor	1.71	0.56-5.30	11.9	59	0.31	0.02-4.57	2.9	34	7.4	74
Church denomination										
Protestant	1.10	0.80-1.51	9.5	872	1.12	0.80-1.57	9.4	769	8.8	1,015
Roman Catholic	0.64	0.36-1.13	7.9	277	0.59	0.34-1.01	7.3	302	11.5	353
Pentecostal	0.94	0.70-1.25	10.0	1,029	0.76	0.56-1.04	8.5	933	11.3	1,153
Apostolic	1.17	0.92-1.48	13.4	1,377	0.96	0.74-1.25	11.5	1,016	12.4	1,140
Zionist	0.78	0.45-1.35	9.4	256	0.94	0.54-1.64	10.8	222	12.1	324
Other	1.01	0.64-1.60	13.6	258	0.77	0.51-1.15	10.7	506	12.7	551
None	0.83	0.32-2.16	9.9	91	1.51	0.62-3.71	14.1	78	11.5	116
HIV infection status										
Infected - on ART		-				-			7.7	313
Infected - no ART		-				-			6.6	170
Uninfected		-				-			11.8	3,954
Birth/pregnancy history										
Nulli-parous	1.19	0.17-8.16	0.19	1,046	7.76	1.72-35.0	1.1	969	0.14	1,384
Parous	1.10	0.95-1.26	14.7	3,114	0.96	0.82-1.11	12.9	2,857	14.8	3,268

[1]A recent birth was taken to be one that occurred in the year before interview.

AOR: Odds ratios from multivariable logistic regression adjusted for age-group and site type.

Table S2 Adjusted odds ratios for sexual abstinence and consistent use of family planning methods in sexually-experienced women aged 15-49 years by marital status: late- and mid- Covid-19 reports compared to before Covid-19, Manicaland, Zimbabwe

	Sexual abstinence[1]										Consistent use of family planning methods[2]									
	Late-Covid-19 *vs.* Pre-Covid-19				Mid-Covid-19 *vs* . Pre-Covid-19				Pre- Covid-19		Late-Covid-19 *vs.* Pre-Covid-19				Mid-Covid-19 *vs* . Pre-Covid-19				Pre- Covid-19	
Characteristic	aOR	*95% per cent CI*	%	*N*	aOR	*95% per cent CI*	%	*N*	%	*N*	aOR	*95% per cent CI*	%	*N*	aOR	*95% per cent CI*	%	*N*	%	*N*
Sexually-experienced women																				
All	1.31	1.17-1.47	20.4	3381	1.27	1.14-1.42	19.9	3102	16.6	3679	1.05	0.96-1.16	63.9	3381	0.82	0.74-0.90	58.8	3102	62.1	3679
Single	1.66	1.13-2.44	54.2	192	1.77	1.19-2.64	56.7	157	44.1	295	0.94	0.62-1.42	33.3	192	0.80	0.52-1.24	30.6	157	36.8	295
Married	1.51	1.22-1.87	7.7	2562	1.42	1.14-1.77	7.1	2388	5.2	2803	0.97	0.87-1.09	69.9	2562	0.74	0.66-0.83	64.6	2388	69.2	2803
Divorced or separated	1.31	1.01-1.69	60.5	479	1.35	1.03-1.76	61.1	409	54.9	428	1.21	0.94-1.57	52.6	479	0.96	0.73-1.25	46.7	409	47.5	428
Widowed	1.32	0.81-2.14	67.6	148	1.60	0.99-2.58	73.0	148	63.4	153	2.08	1.23-3.52	37.2	148	1.46	0.90-2.37	28.4	148	22.0	153
Married women																				
Age-group																				
15-19	0.62	0.28-1.34	7.0	128	1.35	0.73-2.50	15.2	112	12.1	273	0.72	0.46-1.11	37.5	128	0.71	0.45-1.12	36.6	112	44.7	273
20-24	1.27	0.75-2.13	6.8	400	2.09	1.29-3.39	9.9	354	5.3	685	0.73	0.56-0.93	58.0	400	0.57	0.44-0.74	52.5	354	66.0	685
25-34	2.59	1.63-4.11	7.0	829	1.63	0.99-2.66	4.6	828	2.9	908	0.96	0.78-1.19	73.7	829	0.65	0.53-0.80	65.8	828	74.6	908
35-49	1.46	1.06-2.02	8.5	1205	1.23	0.88-1.70	7.2	1094	6.0	937	1.26	1.05-1.51	74.7	1205	1.00	0.83-1.20	70.4	1094	70.3	937
Location																				
Urban	1.55	1.02-2.37	9.1	617	1.53	0.98-2.38	9.4	416	7.6	526	1.11	0.87-1.42	67.1	617	0.87	0.67-1.12	60.8	416	62.0	526
Periurban	2.14	1.36-3.37	7.6	710	1.69	1.06-2.69	6.0	745	3.7	833	0.94	0.76-1.16	69.7	710	0.85	0.68-1.05	67.5	745	69.7	833
Agricultural estate	1.92	1.18-3.15	7.4	605	1.48	0.88-2.49	5.8	552	3.8	625	0.91	0.71-1.16	70.7	605	0.66	0.52-0.85	64.7	552	71.0	625
Rural	0.99	0.67-1.47	6.7	630	1.21	0.83-1.76	7.9	675	6.8	819	0.96	0.77-1.20	72.1	630	0.63	0.50-0.78	63.6	675	71.9	819

[1]Women reporting not having had sex for 3 months or more were taken to have been abstaining from sexual activity.

[2]Includes modern and traditional methods. Women who reported using a method of contraception most or all of the time were taken to have been using family planning methods consistently.

aOR: Odds ratios from multivariable logistic regression adjusted for age-group and community location.
